# Survival and Safety Outcomes of Three-Cycle Adjuvant Chemotherapy in Intermediate-Risk Endometrial Cancer †

**DOI:** 10.3390/cancers18091380

**Published:** 2026-04-26

**Authors:** Shota Higami, Yasuyuki Kinjo, Tomoko Kurita, Kiyoshi Yoshino

**Affiliations:** Department of Obstetrics and Gynecology, University of Occupational and Environmental Health, Kitakyushu 807-8555, Fukuoka, Japan; s-higami0501@med.uoeh-u.ac.jp (S.H.); kinjo-yasuyuki@med.uoeh-u.ac.jp (Y.K.); k-yoshino@med.uoeh-u.ac.jp (K.Y.)

**Keywords:** endometrial neoplasms, intermediate risk, paclitaxel, carboplatin, disease-free survival

## Abstract

**Objective:** The optimal adjuvant therapy for intermediate-risk endometrial cancer remains controversial. Although radiotherapy is commonly used in Western countries, chemotherapy is preferred in Japan; however, its real-world outcomes remain limited. This study evaluated the survival and safety outcomes of three-cycle adjuvant chemotherapy in patients with intermediate-risk endometrial cancer. **Methods:** We retrospectively analyzed patients who underwent primary surgery for endometrial cancer at a university hospital between 2008 and 2019. Low- and intermediate-risk patients defined by the 2013 Japan Society of Gynecologic Oncology recurrence risk classification were included. Intermediate-risk patients were classified as receiving chemotherapy (Int-Chemo+) or not (Int-Chemo−). The primary endpoint was disease-free survival (DFS); secondary endpoints included cancer-specific survival (CSS), adverse events, and treatment completion. **Results:** Among 232 patients, 161 were low-risk and 71 intermediate-risk; 49 intermediate-risk patients received platinum-based combination chemotherapy. The 5-year DFS rates were 92.8% (95% CI, 88.6–96.9) in the low-risk group, 89.4% (95% CI, 80.7–98.2) in the Int-Chemo+ group, and 73.5% (95% CI, 53.5–93.6) in the Int-Chemo− group (log-rank *p* = 0.005). DFS differed between the Int-Chemo+ and Int-Chemo− groups (HR 0.232, 95% CI 0.062–0.867), whereas the DFS outcomes of the Int-Chemo+ group were numerically similar to those of the low-risk group. CSS did not differ significantly among groups (*p* = 0.052). Treatment completion was 93.8%, and grade ≥ 3 adverse events were mainly hematologic, without severe late toxicities. **Conclusions:** Three cycles of adjuvant platinum-based combination chemotherapy for intermediate-risk patients may be associated with improved DFS, while maintaining a high treatment completion rate and manageable toxicity.

## Simple Summary

The optimal postoperative treatment for intermediate-risk endometrial cancer remains controversial, particularly because treatment strategies differ between Japan and Western countries. In Japan, adjuvant chemotherapy is frequently used, and six-cycle regimens are often adopted in clinical practice. However, evidence regarding the survival and safety outcomes of a three-cycle strategy remains limited. In this retrospective single-institution study, we evaluated survival outcomes and safety in patients with intermediate-risk endometrial cancer classified according to the 2013 JSGO recurrence risk classification. Our results suggest that three cycles of platinum-based adjuvant chemotherapy may be associated with improved disease-free survival while maintaining a high treatment completion rate and manageable toxicity. No severe late toxicities were observed. However, because of the retrospective design, baseline imbalance between groups, and the limited number of recurrence events, these findings should be interpreted cautiously. Overall, this three-cycle strategy may represent a practical treatment option in real-world clinical practice, although further investigation in larger prospective studies is warranted.

## 1. Introduction

Endometrial cancer is one of the most common gynecological malignancies in developed countries, and its incidence has been rising in recent years [1,2]. In Japan, the increasing number of cases is largely attributed to changes in lifestyle and an aging population [3,4]. Although most cases are diagnosed at an early stage, approximately 7% of patients with early-stage disease experience recurrence, which adversely affects prognosis [2]. Therefore, selecting an appropriate adjuvant therapy is essential to reduce recurrence and improve long-term outcomes in early-stage disease.

Previous clinical trials have evaluated adjuvant therapy according to recurrence risk. The Gynecologic Oncology Group (GOG)-99 trial demonstrated the efficacy of external beam radiotherapy (EBRT) in patients with intermediate-risk disease [5]. Subsequently, the Postoperative Radiation Therapy in Endometrial Carcinoma (PORTEC)-2 trial showed that vaginal brachytherapy (VBT) was non-inferior to EBRT and associated with lower toxicity, establishing it as the standard adjuvant treatment [6]. In contrast, the Japanese Gynecologic Oncology Group (JGOG)-2033 trial compared chemotherapy (CAP regimen: cyclophosphamide, doxorubicin, and cisplatin) with pelvic radiotherapy (PRT) and found no significant difference in survival outcomes. However, chemotherapy was associated with a reduced risk of distant recurrence. Consequently, adjuvant chemotherapy has been widely adopted in Japan for both high- and intermediate-risk disease [7]. Subsequently, taxane-platinum regimens became more widely used, and paclitaxel plus carboplatin (TC) has become one of the commonly used postoperative regimens in clinical practice, with favorable feasibility and a manageable toxicity profile [8,9,10].

However, the definition of intermediate risk varies across trials and clinical guidelines (Supplementary Table S1). According to the European guidelines (ESGO–ESTRO–ESP 2021), intermediate risk is subdivided into low-intermediate (LIR) and high-intermediate (HIR). Observation is generally recommended for LIR cases, although VBT may also be used. In contrast, VBT is recommended for HIR, whereas EBRT may be considered for patients at substantial risk of pelvic recurrence [11,12]. In contrast, the 2023 Japanese Society of Gynecologic Oncology (JSGO) guidelines retain the 2013 JSGO recurrence risk classification, defining intermediate-risk as a single category and proposing postoperative chemotherapy as a treatment option [13].

Most previous trials have primarily included patients with HIR or high-risk (HR) disease, mainly evaluating radiotherapy or chemoradiation. Consequently, evidence supporting chemotherapy alone in intermediate-risk patients remains limited. Recent reviews continue to debate the optimal adjuvant therapy, highlighting the variability across institutions and regions [2,14]. Moreover, although six cycles of chemotherapy are commonly used, toxicity and completion rates remain major concerns, and no consensus exists regarding the optimal number of cycles [10]. In our institution, three cycles of adjuvant chemotherapy have been used for patients with intermediate-risk disease in routine clinical practice. Given this background, we considered it clinically meaningful to examine the real-world outcomes of this three-cycle strategy.

Therefore, this study aimed to evaluate the real-world survival and safety outcomes of three cycles of adjuvant chemotherapy in patients with intermediate-risk endometrial cancer, as defined by the 2013 JSGO recurrence risk classification.

This study extends a conference presentation previously reported at the 78th Annual Congress of the Japan Society of Obstetrics and Gynecology [15].

## 2. Materials and Methods

### 2.1. Study Design and Ethical Considerations

This single-institution, retrospective study was approved by the Ethics Committee of Medical Research, University of Occupational and Environmental Health, Japan (Approval No. UOEHCRB21-155). Informed consent was obtained using an opt-out approach. Eligible patients were those diagnosed with endometrial cancer at our institution between January 2008 and December 2019 who underwent hysterectomy.

### 2.2. Patient Population and Risk Classification

Postoperative pathology findings were used to classify patients into low- or intermediate-risk groups according to the 2013 JSGO recurrence risk classification [13]. Low-risk patients were defined as endometrioid carcinoma G1/G2 with <50% myometrial invasion and negative lymphovascular space invasion (LVSI). Intermediate-risk patients were defined as follows: (i) endometrioid carcinoma G1/G2 with ≥50% invasion (regardless of LVSI); (ii) G1/G2 with <50% invasion and positive LVSI; (iii) G3 with <50% invasion (regardless of LVSI); or (iv) serous/clear cell carcinoma without invasion and negative LVSI. Patients with synchronous malignancies were excluded. Patients without lymphadenectomy were included if pre- or postoperative imaging (computed tomography [CT], magnetic resonance imaging [MRI], or positron emission tomography/computed tomography [PET-CT]) showed no lymph node enlargement and were therefore considered clinically node-negative.

### 2.3. Treatment

The choice of regimen, number of cycles, and any dose modifications were determined according to institutional policy and the treating physician’s discretion, considering patient age, comorbidities, and preference. Intermediate-risk patients generally received three cycles of adjuvant chemotherapy. The most commonly used regimen was paclitaxel plus carboplatin (TC) (paclitaxel 180 mg/m^2^ and carboplatin AUC 5). Some patients received docetaxel plus carboplatin (DC) (docetaxel 70 mg/m^2^ and carboplatin AUC 5) or doxorubicin plus cisplatin (AP) (doxorubicin 60 mg/m^2^ and cisplatin 50 mg/m^2^). All regimens were administered every 3 weeks. Patients who received at least one cycle were categorized as the chemotherapy group (Int-Chemo+), and completion was defined as receiving three cycles. Those without chemotherapy were classified as the non-chemotherapy group (Int-Chemo−). Adequate lymphadenectomy was defined as the removal of ≥10 lymph nodes.

### 2.4. Endpoints

The primary endpoint was disease-free survival (DFS), defined as the interval from surgery to recurrence or last follow-up. Secondary endpoints included cancer-specific survival (CSS; defined as surgery to death from endometrial cancer), site of recurrence (local, distant, or nodal), grade ≥ 3 adverse events (per CTCAE v5.0), and treatment completion. Recurrence was confirmed by imaging or pathological examination.

### 2.5. Data Collection

Clinical data included age, body mass index (BMI), FIGO 2008 stage, histology, myometrial invasion (≥50% vs. <50%), LVSI, lymphadenectomy status, chemotherapy details (regimen, number of cycles, reasons for omission), recurrence (presence, timing, and site), cancer-specific death, and grade ≥ 3 adverse events.

### 2.6. Statistical Analysis

Analyses were performed using R version 4.5.1. Continuous variables were summarized as medians with interquartile ranges (IQRs), and categorical variables were presented as numbers and percentages. Patient characteristics across the three groups were summarized descriptively. Within the intermediate-risk cohort, comparisons were made using the Mann–Whitney U test for continuous variables and the chi-square or Fisher’s exact test for categorical variables. Survival outcomes were estimated using the Kaplan–Meier method and compared with the log-rank test. Hazard ratios (HRs) with 95% confidence intervals (CIs) were calculated using Cox proportional hazards models, with the Int-Chemo− group as the reference. Multiplicity was adjusted using the Holm method, and statistical significance was set at *p* < 0.05. Additional sensitivity analyses were performed within the intermediate-risk cohort, including a per-protocol analysis restricted to patients who completed three cycles of chemotherapy, an analysis restricted to patients treated with TC, and an analysis restricted to patients who underwent lymphadenectomy. Given the limited number of recurrence events in the intermediate-risk cohort, a Cox proportional hazards model adjusted for age as a continuous variable was examined as an exploratory analysis.

## 3. Results

### 3.1. Patient Characteristics

**Figure 1:**
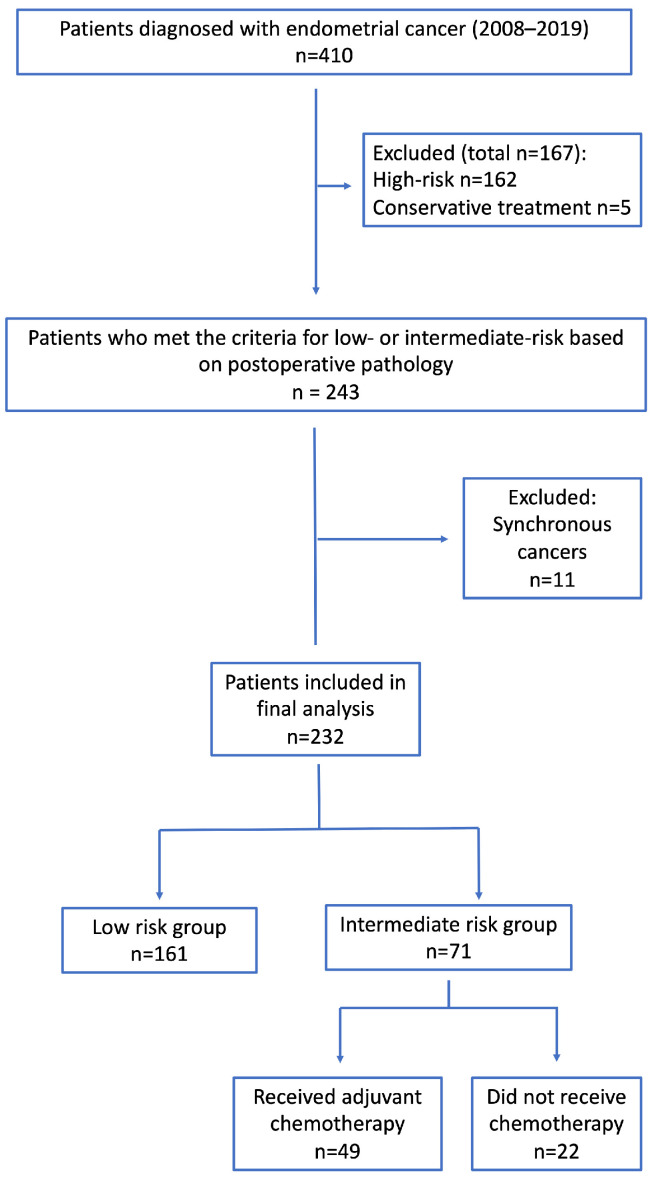
Patient selection and risk stratification. Flow diagram of patient selection. Of 410 patients diagnosed with endometrial cancer between 2008 and 2019, 162 with high-risk pathology and 5 who received conservative treatment were excluded (total n = 167). Among 243 patients classified as low- or intermediate-risk, 11 with synchronous cancers were further excluded, leaving 232 for analysis (low-risk group n = 161; intermediate-risk group n = 71 [adjuvant chemotherapy n = 49; no chemotherapy n = 22]).

Of the 410 patients diagnosed with endometrial cancer between 2008 and 2019, 162 were classified as high-risk based on postoperative pathology, and five who received conservative treatment (total n = 167) were excluded. Among the remaining 243 patients classified as low- or intermediate risk, 11 with synchronous malignancies were excluded, leaving 232 patients for the final analysis. The final cohort comprised 161 low-risk and 71 intermediate-risk patients, of whom 49 received adjuvant chemotherapy (Int-Chemo+), and 22 did not (Int-Chemo−) (Figure 1).

Patient characteristics are summarized in Table 1. Median ages were 57.0 years (IQR 49.0–66.0) for low-risk, 60.0 (IQR 54.0–71.0) for Int-Chemo+, and 77.5 (IQR 70.0–83.5) for Int-Chemo−. BMI and comorbidity rates did not differ significantly between the groups. Adequate lymphadenectomy (≥10 nodes) was less frequent in the Int-Chemo− group. Within the intermediate-risk cohort, Int-Chemo− patients were significantly older and less likely to undergo adequate lymphadenectomy compared with Int-Chemo+ patients (both *p* < 0.001; Supplementary Table S2). No significant differences were observed in comorbidities, FIGO 2008 stage, or pathological factors. Among the 22 Int-Chemo− patients, chemotherapy was omitted due to age (n = 12), comorbidities (n = 6), or patient preference (n = 4).

### 3.2. Disease-Free Survival (DFS; Primary Endpoint)

**Figure 2:**
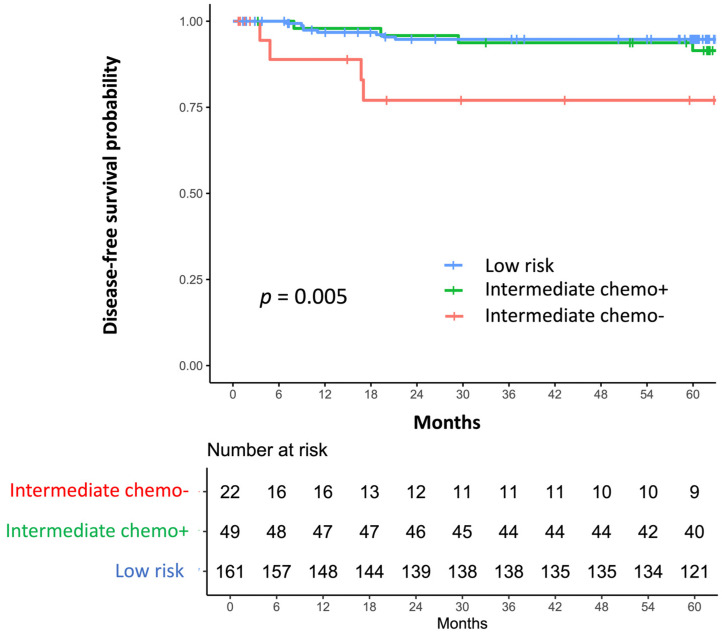
Kaplan–Meier curves for disease-free survival (DFS) by risk group and chemotherapy status. Tick marks indicate censoring; numbers at risk are shown. The survival curves of the low-risk and Int-Chemo+ groups overlapped, whereas the Int-Chemo− group had persistently poorer outcomes (log-rank *p* = 0.005). The 5-year DFS rates were 92.8% (95% CI, 88.6–96.9) in the low-risk group, 89.4% (95% CI, 80.7–98.2) in the Int-Chemo+ group, and 73.5% (95% CI, 53.5–93.6) in the Int-Chemo− group.

Kaplan–Meier analysis showed significant differences in DFS among the groups (log-rank *p* = 0.005; Figure 2). The DFS curves for the low-risk and Int-Chemo+ groups overlapped, whereas the Int-Chemo− group showed poorer outcomes. In pairwise Cox regression analysis (reference = Int-Chemo−; Holm-adjusted), HRs were 0.232 (95% CI 0.062–0.867; *p* = 0.039) for Int-Chemo+ vs. Int-Chemo− and 0.208 (95% CI 0.072–0.601; *p* = 0.005) for low-risk vs. Int-Chemo−. Five-year DFS rates were 92.8% (95% CI 88.6–96.9) in the low-risk group, 89.4% (95% CI 80.7–98.2) in Int-Chemo+, and 73.5% (95% CI 53.5–93.6) in Int-Chemo−. Median follow-up duration was 72.2 months (IQR 60.4–103.0) for low-risk, 80.4 months (IQR 63.5–109.2) for Int-Chemo+, and 51.4 months (IQR 10.6–77.2) for Int-Chemo−.

Additional sensitivity analyses were performed within the intermediate-risk cohort. In the per-protocol analysis restricted to patients who completed three cycles of chemotherapy, the association was similar to that observed in the main analysis (HR 0.189, 95% CI 0.045–0.795; *p* = 0.023; Supplementary Figure S1). Likewise, in the TC-only analysis, the association was similar to that observed in the main analysis (HR 0.204, 95% CI 0.048–0.857; *p* = 0.030; Supplementary Figure S2). In the lymphadenectomy-restricted analysis and the exploratory Cox model adjusted for age, statistical significance was not retained (HR 0.225, 95% CI 0.023–2.167; *p* = 0.197, and HR 0.254, 95% CI 0.058–1.116; *p* = 0.070, respectively).

### 3.3. Cancer-Specific Survival (CSS; Secondary Endpoint)

**Figure 3:**
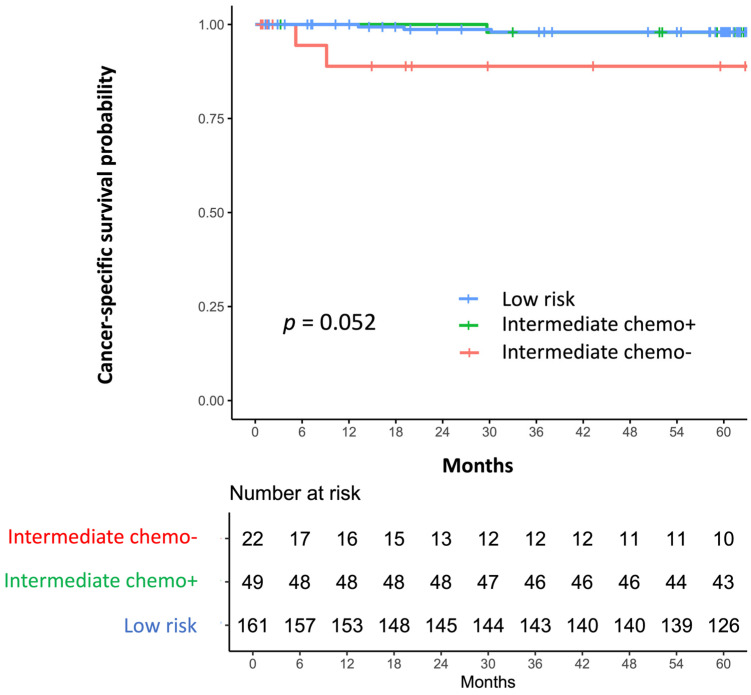
Kaplan–Meier curves for cancer-specific survival (CSS) by risk group and chemotherapy status. Tick marks indicate censoring; numbers at risk are shown. Kaplan–Meier analysis showed no statistically significant differences in CSS among the groups (log-rank *p* = 0.052). The 5-year CSS rates were 98.0% (95% CI, 95.7–100.0) in the low-risk group, 97.9% (95% CI, 93.9–100.0) in the Int-Chemo+ group, and 88.9% (95% CI, 74.7–100.0) in the Int-Chemo− group.

Kaplan–Meier analysis showed no statistically significant differences in CSS among the groups (log-rank *p* = 0.052; Figure 3). Pairwise Cox regression analysis (reference = Int-Chemo−; Holm-adjusted) yielded HRs of 0.288 (95% CI 0.040–2.060; *p* = 0.350) for Int-Chemo+ vs. Int-Chemo− and 0.141 (95% CI 0.023–0.849; *p* = 0.055) for low-risk vs. Int-Chemo−. Five-year CSS rates were 98.0% (95% CI, 95.7–100.0) in the low-risk group, 97.9% (95% CI, 93.9–100.0) in the Int-Chemo+ group, and 88.9% (95% CI, 74.7–100.0) in the Int-Chemo− group. Seven cancer-specific deaths occurred: three (1.9%) in low-risk, two (4.1%) in Int-Chemo+, and two (9.1%) in Int-Chemo−.

### 3.4. Adjuvant Chemotherapy and Adverse Events

Chemotherapy regimens and adverse events are summarized in Table 2. Among the 49 treated patients, 43 (87.8%) received TC, five (10.2%) received DC, and one (2.0%) received AP. A total of 46 patients (93.8%) completed all three planned cycles, one patient (2.0%) received two cycles, and two patients (4.2%) received only one cycle. Grade ≥ 3 adverse events occurred in 25 patients (51.0%): primarily hematologic (22 patients, 44.8%), including neutropenia in 20 (40.8%), anemia in 1 (2.0%), and thrombocytopenia in 1 (2.0%). Non-hematologic events included nausea in 1 (2.0%) and allergic reactions in 2 patients (4.2%). No grade ≥ 3 peripheral neuropathy was observed.

### 3.5. Patterns of Recurrence

Patterns of recurrence are summarized in Table 3. The recurrence rates were 6.8% (11/161) in the low-risk group, 8.2% (4/49) in the Int-Chemo+ group, and 22.7% (5/22) in the Int-Chemo− group. Patients with recurrence at multiple sites were counted at each site. Overall, 20 patients experienced recurrence at 30 sites. The number of patients/sites was 11/15 in the low-risk group, 4/6 in Int-Chemo+, and 5/9 in Int-Chemo−. In the low-risk group, recurrences included 6 local sites (40.0%; vaginal, n = 3; pelvic, n = 3), 5 distant sites (33.3%; liver, n = 2; lung, n = 2; peritoneal dissemination, n = 1), and 4 nodal sites (26.7%; pelvic, n = 1; para-aortic, n = 3). In the Int-Chemo+ group, 3 sites (50.0%) were local (vaginal, n = 1; pelvic, n = 2), 1 site (16.7%) was distant (lung), and 2 sites (33.3%) were nodal (pelvic, n = 2). In the Int-Chemo− group, 5 sites (55.6%) were local (vaginal, n = 3; pelvic, n = 2), 3 sites (33.3%) were distant (lung, n = 2; bone, n = 1), and 1 site (11.1%) was nodal (pelvic).

## 4. Discussion

This study compared low-risk, Int-Chemo+, and Int-Chemo− groups to evaluate the survival and safety outcomes of platinum-based adjuvant chemotherapy in intermediate-risk endometrial cancer. DFS differed significantly among the groups, with poorer outcomes in the Int-Chemo− group, whereas the low-risk and Int-Chemo+ groups showed numerically similar DFS. In contrast, CSS did not differ significantly. Adjuvant chemotherapy was associated with a high completion rate of the planned three-cycle regimen, and most grade ≥ 3 adverse events were manageable hematologic toxicities. In this retrospective cohort, three cycles of adjuvant chemotherapy may be associated with improved DFS in intermediate-risk patients while maintaining a high treatment completion rate and manageable toxicity. However, because of the marked baseline imbalance between the Int-Chemo+ and Int-Chemo− groups, particularly with respect to age and lymphadenectomy status, these findings should be interpreted cautiously.

In Western clinical trials, such as GOG-99 and PORTEC, risk classification is based on age, FIGO stage, histology, and lymphovascular space invasion (LVSI). For patients with LIR and HIR, radiotherapy, particularly VBT, is recommended as the standard adjuvant treatment [5,6]. In Japan, the JGOG2033 trial compared chemotherapy with radiotherapy and found no significant differences in survival rates. However, chemotherapy was associated with a reduced risk of distant recurrence. Therefore, adjuvant chemotherapy has been widely adopted in Japan for patients with high- and intermediate-risk disease [7]. However, these trials primarily targeted HIR or high-risk disease, and evidence regarding the survival outcomes, safety, and optimal number of chemotherapy cycles for intermediate-risk patients remains limited.

Previous studies investigating adjuvant chemotherapy for intermediate-risk endometrial cancer have yielded conflicting results. A prospective study in HIR patients (aged ≥ 60 years or with two or more risk factors: grade 3 histology, LVSI, or myometrial invasion ≥ 50%) demonstrated that three cycles of platinum-based chemotherapy were effective in preventing recurrence [16]. Another prospective study that included patients classified as HIR according to GOG-99 suggested that adjuvant chemotherapy may provide progression-free survival (PFS) and overall survival (OS) outcomes comparable to those of radiotherapy [17]. In contrast, a large Japanese retrospective study evaluated stage I–II, node-negative patients after lymphadenectomy and stratified them according to the 2013 JSGO recurrence risk classification [18]. In that study, no significant differences in DFS or OS were observed between patients who did and did not receive adjuvant chemotherapy in either risk subgroup. These inconsistent results may reflect differences in risk classification criteria, patient characteristics, and treatment intensity across studies.

In our study, we evaluated the clinical outcomes of three-cycle adjuvant chemotherapy in patients classified as intermediate risk according to the 2013 JSGO recurrence risk classification, including those in whom lymphadenectomy was not performed. We observed an association between adjuvant chemotherapy and DFS, whereas CSS did not differ significantly between the groups. In early-stage endometrial cancer, event rates are low, and outcomes are influenced by post-recurrence treatments and competing non-cancer mortality, making differences in CSS or OS difficult to detect [5]. In addition, the intermediate-risk category used in this study included patients who would have been classified as LIR in other studies, which may have lowered the overall baseline recurrence risk of the cohort [5,6,18]. More importantly, the marked baseline imbalance between the Int-Chemo+ and Int-Chemo− groups represents the major limitation in interpreting the DFS difference. In the Int-Chemo− group, omission of chemotherapy was related to advanced age or comorbidities, suggesting selection bias, while omission of lymphadenectomy may have increased the risk of understaging. Therefore, the observed DFS difference may not be attributable to chemotherapy alone and should be interpreted cautiously.

Ideally, comprehensive multivariable adjustment would have been performed to better account for baseline imbalance and potential confounding. However, because only 9 recurrence events occurred in the intermediate-risk cohort, stable multivariable modeling incorporating multiple clinicopathologic variables was not feasible. Therefore, to further explore the potential impact of baseline imbalance and understaging, we performed a lymphadenectomy-restricted analysis and a simple age-adjusted Cox model as exploratory analyses. In the lymphadenectomy-restricted analysis, only 5 patients remained in the Int-Chemo− group, substantially limiting interpretation. Likewise, the age-adjusted Cox model was considered exploratory because of the limited number of recurrence events. Although statistical significance was not retained, the point estimates in these analyses remained in the same direction as the main analysis (HR 0.225 in the lymphadenectomy-restricted analysis and HR 0.254 in the age-adjusted model, compared with HR 0.232 in the main analysis). However, these analyses do not eliminate residual confounding, and the findings should still be interpreted as exploratory rather than causal.

Nonetheless, in real-world clinical practice, adequate staging surgery is often not feasible in elderly patients or those with substantial perioperative risks. In this context, it is of interest that DFS in the Int-Chemo+ group was numerically similar to that of the low-risk group, which is generally expected to have a lower baseline risk of recurrence. Although this study applied the 2013 JSGO intermediate-risk classification, which differs from international criteria, the results reflect real-world clinical practice in Japan and provide insights into regional differences in treatment strategies.

In clinical practice, treatment decisions should consider not only survival outcomes but also treatment completion and toxicity. In this study, three cycles of adjuvant chemotherapy were administered with a high completion rate and acceptable toxicity. In addition, sensitivity analyses restricted to patients who completed all three cycles or received the TC regimen showed a similar direction of DFS association. These findings suggest that a three-cycle strategy may be a practical postoperative option in intermediate-risk endometrial cancer.

In Western countries, VBT has become the standard adjuvant treatment for intermediate-risk patients based on the results of the PORTEC-II trial [6]. Compared with EBRT, VBT is associated with fewer acute gastrointestinal and genitourinary toxicities and is generally considered safer [19]. However, late toxicities, such as vaginal atrophy and stenosis, which can impair sexual function, as well as urinary symptoms, including frequency and incontinence, have been reported with an incidence rate of 15–25% [20,21]. Although these adverse effects are not directly life-threatening, they may have long-term impacts on sexual activity and quality of life (QOL), representing an important clinical consideration.

In contrast, the adverse effects of chemotherapy are primarily acute hematologic toxicities, which may compromise treatment completion [22]. In the present study, grade ≥ 3 adverse events occurred in 51% of patients receiving three cycles of adjuvant chemotherapy, of which 88% were hematologic toxicities (44.8% overall). These toxicities were manageable with supportive care, and the treatment completion rate was 93.8%.

Although the study population and chemotherapy regimens differed, the JGOG 2043 trial compared six cycles of doxorubicin + cisplatin (AP), docetaxel + cisplatin (DP), and paclitaxel + carboplatin (TC) and reported lower completion rates (AP, 79.8%; DP, 82.9%; TC, 76.0%). In that trial, grade 3/4 hematologic toxicities were predominantly neutropenia, occurring at high frequencies across all regimens (AP, 96.6%; DP, 89.3%; TC, 91.5%) [10], indicating that such toxicities can be major obstacles to treatment continuation. In contrast, Gao et al. reported that limiting adjuvant chemotherapy to three cycles in patients with HIR disease allowed all patients to complete therapy [16]. Although differences in patient backgrounds and supportive care systems must be considered, these findings suggest that optimizing the number of chemotherapy cycles may improve both safety and treatment completion rates.

In the present study, 87.8% of patients received the TC regimen. Peripheral neuropathy associated with paclitaxel is strongly dose-dependent, with the risk increasing sharply once the cumulative dose reaches approximately 1000 mg/m^2^ [23]. In Japan, the standard paclitaxel dose for endometrial cancer is 180 mg/m^2^ per cycle. Accordingly, the cumulative dose is 540 mg/m^2^ after three cycles and 1080 mg/m^2^ after six cycles, exceeding the reported threshold for neuropathy. In fact, peripheral neuropathy was reported in 6.1% of patients in the JGOG2043 study, in which six cycles of chemotherapy, including a paclitaxel plus carboplatin regimen, were administered [10]. In the present study, limiting treatment to three cycles was not associated with any cases of grade ≥ 3 peripheral neuropathy. Taken together, these findings suggest that three cycles of adjuvant TC chemotherapy may represent a practical approach that balances treatment feasibility and safety in intermediate-risk endometrial cancer.

In this study, the proportion of distant recurrences appeared to be lower in the Int-Chemo+ group than in the low-risk and Int-Chemo− groups. Moreover, despite the higher frequency of lymphadenectomy in the Int-Chemo+ group, pelvic recurrences, including nodal relapses, remained relatively common. Furthermore, even among patients classified as low risk, a notable proportion of recurrences involved distant metastases, which contrasts with the general expectation that local recurrence predominates in this group [24,25]. These observations suggest that conventional clinicopathological risk classification alone may not fully capture biological heterogeneity [26,27]. However, because the number of recurrence events was very small (n = 20), these findings should be regarded as exploratory and hypothesis-generating. Further studies are needed to clarify whether patterns of distant and locoregional recurrence differ according to adjuvant treatment strategy and underlying tumor biology.

In recent years, risk stratification based on molecular classification, as proposed by The Cancer Genome Atlas (TCGA), has attracted increasing attention [28,29]. Approximately 5% of cases in the low-risk group exhibit the p53-abnormal subtype (high copy number), which is associated with poor prognosis [30]. In addition, recurrence patterns differ across molecular subtypes: dMMR tumors are prone to local recurrence, NSMP tumors to distant recurrence, and p53-abnormal tumors to intraperitoneal relapse [31]. In the present study, molecular classification was not performed; therefore, the biological basis of recurrence patterns could not be directly assessed. These considerations highlight the importance of future studies integrating molecular classification with conventional clinicopathological factors to achieve more refined risk stratification and more individualized adjuvant treatment strategies.

This study has several limitations. First, it was a retrospective analysis conducted at a single institution; therefore, the influence of selection bias on treatment decisions and patient backgrounds could not be completely eliminated. Second, the sample size was limited to 232 patients, with a particularly small number of patients with intermediate-risk disease, which may have reduced the statistical power of the analysis. Therefore, stable multivariable modeling incorporating multiple clinical and pathological variables was not feasible because of the limited number of events. Third, some patients did not undergo lymphadenectomy because of advanced age or comorbidities and were considered node-negative based on imaging findings, raising the possibility of understaging and stage migration bias. Fourth, molecular classification (such as the TCGA system) was not evaluated in this study; thus, the biological heterogeneity of tumors could not be fully considered. Despite these limitations, this study provides real-world data on the feasibility and safety of a three-cycle strategy and may suggest a possible association with improved DFS in patients with intermediate-risk disease. Future investigations should include multicenter prospective studies with refined risk stratification incorporating molecular classification.

## 5. Conclusions

Three cycles of adjuvant platinum-based combination chemotherapy for patients classified as intermediate risk according to the 2013 JSGO recurrence risk classification may be associated with improved DFS. The treatment completion rate was high, and most adverse events were manageable with supportive care. No severe late toxicities were observed during follow-up, suggesting that this strategy may be a practical option in real-world clinical practice with acceptable tolerability.

However, because of the retrospective design, baseline imbalance between groups, and the limited number of recurrence events, these findings should be interpreted cautiously. Further studies are needed to define the optimal adjuvant treatment strategy in intermediate-risk endometrial cancer, with consideration of recurrence patterns and molecular classification.

## Figures and Tables

**Table 1:** Baseline characteristics of patients with low-risk and intermediate-risk endometrial cancer (Chemotherapy+ vs. Chemotherapy−).

		Low Risk (n = 161)	Intermediate Risk (n = 71)	
			Chemotherapy+ (n = 49)	Chemotherapy− (n = 22)
Median age, years (IQR)		57.0 [49.0–66.0]	60.0 [54.0–71.0]	77.5 [70.0–83.5]
Median BMI, kg/m^2^ (IQR)		23.6 [20.6–28.6]	23.8 [20.8–26.4]	23.7 [19.3–26.3]
Comorbidity		54 (33.5%)	16 (32.7%)	9 (40.9%)
FIGO stage 2008				
	IA	161 (100.0%)	21 (42.9%)	5 (22.7%)
	IB	0 (0.0%)	28 (57.1%)	17 (77.3%)
Histology				
	Endometrioid G1/G2	140 (87.0%)	24 (49.0%)	11 (50.0%)
	Endometrioid G3	21 (13.0%)	16 (32.7%)	9 (40.9%)
	Serous or clear cell carcinoma	0 (0.0%)	9 (18.4%)	2 (9.1%)
Myometrial invasion (≥1/2)				
	No	161 (100.0%)	21 (42.9%)	5 (22.7%)
	Yes	0 (0.0%)	28 (57.1%)	17 (77.3%)
Lymphovascular space invasion				
	No	161 (100.0%)	21 (42.9%)	9 (40.9%)
	Yes	0 (0.0%)	28 (57.1%)	13 (59.1%)
Peritoneal cytology				
	No	146 (90.7%)	42 (85.7%)	21 (95.5%)
	Yes	13 (8.1%)	7 (14.3%)	1 (4.5%)
Surgical method for hysterectomy				
	Laparotomy	108 (67.1%)	44 (89.8%)	21 (95.5%)
	Laparoscopy or Robot-assisted	52 (32.3%)	5 (10.2%)	1 (4.5%)
	Transvaginal	1 (0.6%)	0 (0.0%)	0 (0.0%)
Lymphadenectomy				
	None	42 (26.1%)	4 (8.2%)	17 (77.3%)
	Pelvic lymphadenectomy	116 (72.0%)	41 (83.6%)	5 (22.7%)
	Pelvic and paraaortic lymphadenectomy	3 (1.9%)	2 (4.1%)	0 (0.0%)

Data are median [IQR] or n (%). IQR, interquartile range; BMI, body mass index. Comorbidity = hypertension, diabetes mellitus, dyslipidemia, cerebrovascular disease, heart disease, or renal disease. Lymphadenectomy = removal of ≥10 lymph nodes.

**Table 2:** Chemotherapy regimens, treatment cycles, and grade ≥ 3 adverse events in the intermediate-risk chemotherapy group.

Number of patients		49
Regimen		
	TC	43 (87.8%)
	DC	5 (10.2%)
	AP	1 (2.0%)
Total cycles		
	1	2 (4.2%)
	2	1 (2.0%)
	3	46 (93.8%)
Adverse events (≥Grade 3)		
	No	24 (49.0%)
	Yes	25 (51.0%)
	Neutropenia	20 (40.8%)
	Anemia	1 (2.0%)
	Thrombocytopenia	1 (2.0%)
	Nausea	1 (2.0%)
	Allergy	2 (4.2%)

Data are shown as n (%). Regimens: TC, paclitaxel plus carboplatin; DC, docetaxel plus carboplatin; AP, doxorubicin plus cisplatin. Adverse events were graded according to the Common Terminology Criteria for Adverse Events (CTCAE), version 5.0.

**Table 3:** Patterns of recurrence in patients with low- and intermediate-risk endometrial cancer stratified by adjuvant chemotherapy status.

		Low Risk (n = 161)	Intermediate Risk	
			Chemotherapy+ (n = 49)	Chemotherapy− (n = 22)
Total number of recurrent patients, n (%)		11 (6.8%)	4 (8.2%)	5 (22.7%)
Total number of recurrence sites, n		15	6	9
Local recurrence		6	3	5
	Vagina	3	1	3
	Intrapelvic	3	2	2
Distant recurrence		5	1	3
	Liver	2	0	0
	Lung	2	1	2
	Bone	0	0	1
	Peritoneal dissemination	1	0	0
Node recurrence		4	2	1
	Pelvic lymph node	1	2	1
	Para-aortic lymph node	3	0	0
Proportion within each group (%)		Local 40.0%/Distant 33.3%/Node 26.7%	Local 50.0%/Distant 16.7%/Node 33.3%	Local 55.6%/Distant 33.3%/Node 11.1%

Data are presented as the number of patients and recurrence sites (n). Recurrence rates (%) were calculated based on the total number of patients in each group. Patients with multiple recurrence sites were counted for each site. Proportions of local, distant, and nodal recurrences within each group are summarized at the bottom of the table.

## Data Availability

The data presented in this study are available on request from the corresponding author due to ethical restrictions and patient confidentiality.

## Supplementary Materials

The following supporting information can be downloaded at: https://www.mdpi.com/article/10.3390/cancers18091380/s1, Figure S1: Per-protocol analysis restricted to patients who completed three cycles of chemotherapy; Figure S2: TC-only analysis within the intermediate-risk cohort; Table S1: Comparison of risk classification systems; Table S2: Detailed patient characteristics of the intermediate-risk cohort.
