# Supplementary material for: Survival and Safety Outcomes of Three-Cycle Adjuvant Chemotherapy in Intermediate-Risk Endometrial Cancer
Source: Cancers (Basel). 2026 Apr 26;18(9):1380. doi: 10.3390/cancers18091380 (PMC13162638; doi:10.3390/cancers18091380)
Supplement: Supplementary file 1 [file cancers-18-01380-s001.zip › cancers-4257225-supplementary.pdf]

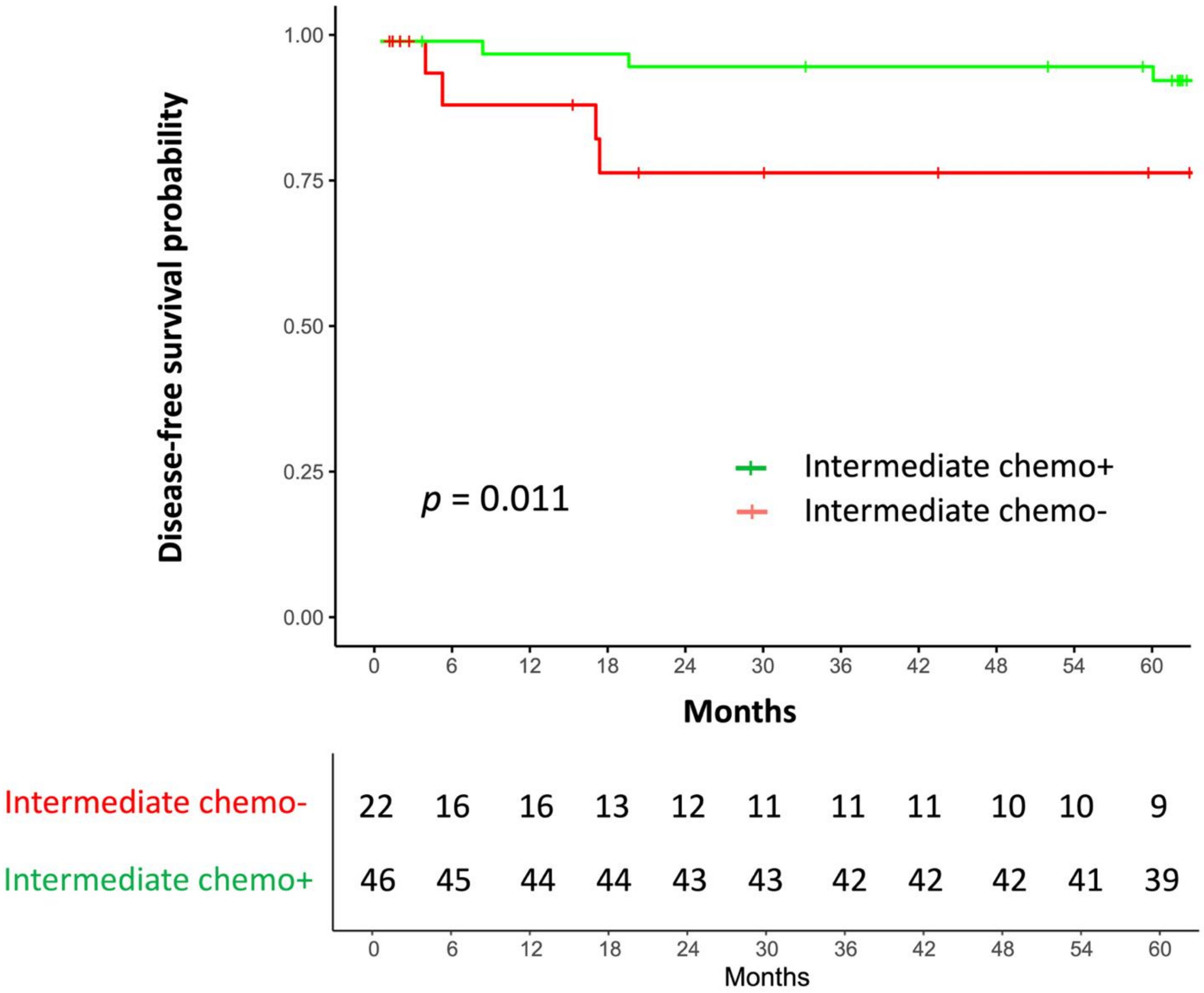

Supplementary Figure S1. Kaplan–Meier curves for disease-free survival in the per-protocol analysis restricted to patients who completed all three planned cycles of chemotherapy. The Int-Chemo+ (3 cycles) group was compared with the Int-Chemo－ group within the intermediate-risk cohort. The p value was calculated using the log-rank test (p = 0.011). Tick marks indicate censoring, and numbers at risk are shown below the plot.

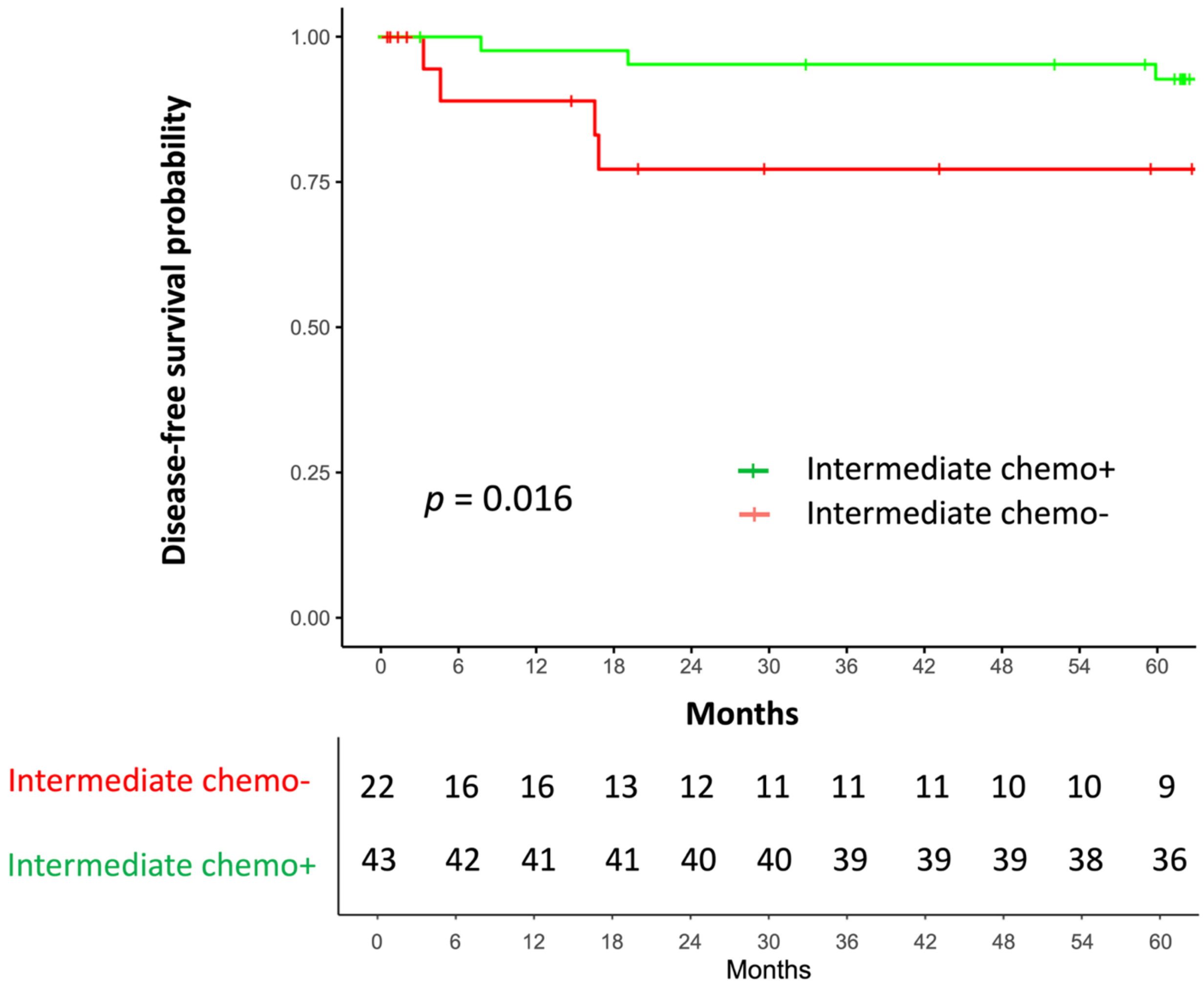

Supplementary Figure S2. Kaplan–Meier curves for disease-free survival in the analysis restricted to patients treated with paclitaxel plus carboplatin (TC) within the intermediate-risk cohort. The Int-Chemo+ (TC-only) group was compared with the Int-Chemo− group. The p value was calculated using the log-rank test (p = 0.016). Tick marks indicate censoring, and numbers at risk are shown below the plot.

Supplementary Table S1:
Comparison of risk group definitions across major guidelines and clinical trials (JSGO 2023, ESGO-ESTRO-ESP 2021, GOG-99, PORTEC I and II)

Guidelines / Clinical trial	Low-intermediate risk	High-intermediate risk
**JSGO 2023**	• Endometrioid G1–2 with ≥50% myometrial invasion (LVSI ±)	
	• Endometrioid G1–2 with <50% myometrial invasion and LVSI +	
	• Endometrioid G3 with <50% myometrial invasion (LVSI ±)	
	• Serous/clear cell carcinoma without myometrial invasion and LVSI−	
**ESGO-ESTRO-ESP 2021** (molecular classification unknown)	• Stage IB endometrioid + low-grade‡ + LVSI negative or focal	• Stage I endometrioid + substantial LVSI regardless of grade and depth of invasion
	• Stage IA endometrioid + high-grade‡ + LVSI negative or focal	• Stage IB endometrioid high-grade‡ regardless of LVSI status
	• Stage IA non-endometrioid (serous, clear cell, undifferentiated carcinoma, carcinosarcoma, mixed) without myometrial invasion	• Stage II
**GOG-99**	• ≤50 years and ≤2 prognostic risk factors (PRFs)	• Any age with 3 PRFs
	• 50–69 years and ≤1 PRF	• 50–69 years and ≥2 PRFs
	• ≥70 years and no PRF	• ≥70 years and ≥1 PRF
**PORTEC I**	Stage I endometrioid carcinoma+	At least 2 of the following 3 factors:
	• G1 and ≥50% MI	• Age >60 years
	• G2	• G3
	• G3 and <50% MI	• ≥50% MI
**PORTEC II**	–	• Age >60 years with Stage IC + G1–2
		• Stage IB + G3
		• Stage IIA (excluded if >50% MI + G3)

Abbreviations: JSGO, Japan Society of Gynecologic Oncology; ESGO, European Society of Gynecological Oncology; ESTRO, European Society for Radiotherapy and Oncology; ESP, European Society of Pathology; GOG, Gynecologic Oncology Group; PORTEC, Post-operative Radiation Therapy in Endometrial Carcinoma; LVSI, lymphovascular space invasion; MI, myometrial invasion; PRF(s), prognostic risk factor(s) per GOG-99—grade 2–3 endometrioid histology, LVSI positive, and outer-third myometrial invasion (deep MI).

‡ According to the binary FIGO grading, grade 1 and grade 2 carcinomas are classified as low-grade, and grade 3 carcinomas as high-grade.

Supplementary Table S2: Baseline clinicopathologic characteristics of intermediate-risk patients stratified by adjuvant chemotherapy status				
		Intermediate risk (n=71)		
		Chemotherapy + (n=49)	Chemotherapy - (n=22)	P value
Median age, years (IQR)		60.0 [54.0-71.0]	77.5 [70.0-83.5]	**<0.001**
Median BMI, kg/m^2(IQR)		23.8 [20.8-26.4]	23.7 [19.3-26.3]	0.872
Comorbidity		16 (32.7%)	9 (40.9%)	0.686
FIGO stage 2008				0.173
	IA	21 (42.9%)	5 (22.7%)	
	IB	28 (57.1%)	17 (77.3%)	
Histology				0.565
	Endometrioid G1/G2	24 (49.0%)	11 (50.0%)	
	Endometrioid G3	16 (32.7%)	9 (40.9%)	
	Serous or clear cell carcinoma	9 (18.4%)	2 (9.1%)	
Myometrial invasion (≥1/2)				0.173
	No	21 (42.9%)	5 (22.7%)	
	Yes	28 (57.1%)	17 (77.3%)	
Lymphovascular space invasion				1.000
	No	21 (42.9%)	9 (40.9%)	
	Yes	28 (57.1%)	13 (59.1%)	
Peritoneal cytology				0.427
	No	42 (85.7%)	21 (95.5%)	
	Yes	7 (14.3%)	1 (4.5%)	
Surgical method for hysterectomy				0.740
	Laparotomy	44 (89.8%)	21 (95.5%)	
	Laparoscopy or Robot-assisted	5 (10.2%)	1 (4.5%)	
	Transvaginal	0 (0.0%)	0 (0.0%)	
Lymphadenectomy				**<0.001**
	None	4 (8.2%)	17 (77.3%)	
	Pelvic lymphadenectomy	41 (83.6%)	5 (22.7%)	
	Pelvic and paraaortic lymphadenectomy	2 (4.1%)	0 (0.0%)	

Data are presented as median [IQR] or n (%).

BMI, body mass index; FIGO stage, 2008 classification.
